# Surgical Eccentricities, or New Memoirs for Reforming and Perfecting Operative Medicine

**Published:** 1845-10

**Authors:** 


### Art. XV.

*Excentricités Chirurgicales, ou Nouveaux Mémoires pour servir à la réforme et au perfectionnement de la Médecine Opératoire.* Par Mathias Mayor.—*Lausanne,* 1844.

*Surgical Eccentricities, or New Memoirs for reforming and perfecting Operative Medicine.* By Mathias Mayor.—*Lausanne,* 1844. 8vo, pp. 420.

This is a would-be-quaint emanation from an egotistical French brain; not calculated, we fear, to achieve the improving and perfecting of the Ars Chirurgica so mightily as the author fondly imagines.

There are fifteen memoirs in this book, all purporting to be very original and very important. The first is entitled "Experience unmasked;" and M. Mayor begins by telling us that, though at the first blush it may seem an ungenerous and perhaps unwarrantable task to take liberties with so grave and venerable a personage as Experience, yet that after all it is no more than right and proper; seeing that he had announced to the scientific Congress of Angers, several years ago, that Experience in Surgery, whatever it may be in other matters, is a nuisance and a mischief, and should be put down—a humbug, and ought to be exposed. The word, it seems, is wrong, because its meaning cannot be accurately defined, and is not generally agreed upon; therefore it ought to be abolished. Much in the same way, we have heard an ingenious friend of ours labour hard to show that the term "Inflammation," is more than ripe for change. But without any pretensions to the absolutely prophetic, we would venture to foretell that both Experience and Inflammation will continue for ages yet to come.

Into an unprofitable discussion on the meaning of words, we have no intention to follow the author. Experience, used as a ground for Empiricism in the healing art, is certainly a thing most wrong and dangerous, and deserves all the abuse we find here heaped upon it. But Experience, used as it ought to be,—and every intelligent practitioner is presumed to know how—is by most men held to be of no inconsiderable value. Why, if, according to M. Mayor's own showing, Experience had done nothing more than tell us that "sulphur cures the itch," the boon were surely worth receiving, and being thankful for, withal. Loud and blustering though M. Mayor is in the expression of his dislike to "Experience in Surgery," may we hope that he will not quarrel with us for venturing to state our belief, that he would be no worse, as man, surgeon, or author, did he possess a little more of that despicable material; unless, indeed, he wish to class himself among those to whom experience teaches nothing.

The second chapter is entitled, "A Sketch of the Principles of Surgery, regarded as a Science and Art." The memoir opens with the general affirmation, that although the treatises on Surgery are voluminous and many, and for the most part written by justly celebrated men, still it is too true that one and all of them leave much for us to desire. M. Mayor declares it to be a matter of the last importance to forearm readers, especially students, against the prodigious activity of authors, which, instead of advancing the interests of science, tends only to perpetuate errors which have already acquired too deep a root. We might here remind M. Mayor, that he too has written a book, and suggest to him, that although its tendency be not to perpetuate old errors, it may possibly have the effect of creating new ones. But we pass from this to express our agreement in his statement, how that a man must be well grounded in the principles of science before he can, with any chance of advantage, occupy himself in promiscuous reading. He must have the means of distinguishing between sense and foolishness, justness and fallacy, truth and error. And these means are the product of a sound education, enhanced by a well-formed habit of thinking. Such readers alone should peruse the work of M. Mayor. To all others it is obviously unsafe.

Having made the general observation that the literature of Surgery is large, and noticed that there are many errors therein contained, M. Mayor boldly tells us, that to discover these errors and root them out, is the object which he has in view. And forthwith he sets to work with a ten-toothed instrument of extirpation, that is, he considers this subject under ten heads. In the first place he most decidedly objects to the use of the phrase "Chirurgie médicale," for, says he, if there is a "Chirurgie médicale," then there must be a "Médecine chirurgicale," and this because the physician bleeds in pneumonia, applies the moxa in sciatica, and prescribes cupping in cases of cerebral congestion. All this, according to M. Mayor, is stuff and nonsense. He reforms by using the adjective "*mécanique;*" for when *it* is associated with the word "médecine," then "tout à coup," we have Surgery proper! "La médecine mécanique" (Surgery proper,) is a very different thing from "la chirurgie médicale," and thoroughly distinct from "la médecine," properly so called! This will serve for a sample of the author's efforts in the "improvement" department. And the following may be taken as a favorable specimen of the modest and becoming spirit in which he is pleased to speak of his own labours: "I enter on my subject," says he, "with the sweet prospect of making, by what I shall say in regard to operative medicine, a great and favorable sensation, and of affording an example of the true way of viewing, studying, comprehending, and criticising all other medicine or medications, however multiplied, varied, eccentric, complicated, or pretending they may be."

At the end of eight pages he finds himself in a condition to define the word Surgery, thus: "it is the science which tends to prevent, cure, or alleviate disease by the employment of mechanical means." This constant harping on *mechanics,* with an apparent wishfulness to tie us down to them, is surely a movement to the rear; tending to bring us back to the olden time when our profession was purely one of handicraft. From such reforms, we hope to be delivered, by the continued prevalence of enlightenment and common sense over bigotry and egotism.

The third chapter treats of the dressing of sores. M. Mayor finds fault with all plans but his own. And his is by the application of "impermeable dressings;" the virtue of which is obviously attributable to the compression which they exert. In this country the profession is already sufficiently alive to the efficacy of treating *certain* sores by compression. Accordingly we refrain from entering further upon this topic; even although M. Mayor tempts us largely, by informing us how he has approached the subject fully equipped; with the aid of "logic and reason, art and the means it supplies, principles and the mode of applying them, observations and facts, analogy and induction, theory and practice, free examination and great good sense!"

The fourth chapter treats of "Tachytomie," or the art of cutting rapidly. Believing as we do, thoroughly, and maintaining still, as we have always done, that surgery is not the mere mechanical art that M. Mayor, and some others, would have it to be, and that the *tuto* is infinitely preferable to the *celeriter* in all cases in which the use of sharp instruments is required, we will readily be excused, no doubt, from further consideration of this topic. Contenting ourselves with simply stating, that in our opinion, M. Mayor has done less than nothing to advance true surgery, by lending the weight of his authority, *quantum valeat,* to move that art in a sinister direction. There is to be found from time to time decided evidence of a leaning towards, if not a fondness for, a worse than meretricious display of doubtful dexterity in the rapid performance of operations, more especially among the junior members of our profession; against this, as opposed to science, surgery, and sense, we have ever set our face strenuously; and we protest solemnly against any countenance being shown to such pseudo-chirurgery, come from what quarter it may. We have the worst opinion of those who are ever inventing new cutting instruments, and vaunting of rapidity and skilfulness in using them. Such, however, is the theme of M. Mayor in this chapter; and we can only say that we profoundly regret that it is so.

The eighth chapter is devoted to "Calomel and Cotton, considered as topical applications in Ophthalmia." "Whoever has been at Lausanne and seen how the dressing department is conducted in the hospital there, or whoever has cast his eye over my works, must be aware that I make extensive use of cotton and wadding." Having given a list of the cases in the treatment of which he employs these substances, and detailed the steps by which he came to use the former in the treatment of ophthalmia, he says,

"The good effects of this precious vegetable production in a considerable number of irritative and inflammatory affections, left no doubt in my mind as to its being possessed of *contrastimulant* properties, and consequently that it would prove an efficacious remedy for analogous diseases situated in or around the eye. I have twenty times caused oculists and other men of art to feel a kind of anxiety, by placing a tompion of cotton between my eye and eyelid, and retaining it there for several seconds. Great was their surprise, however, on remarking that the proceeding did not even give rise to lachrymation."

Being thoroughly assured on this head, and being likewise aware of the modifying power of calomel in inflammations in general, and in affections of the mucous membranes in particular, M. Mayor thought he could not do better than associate the calomel with the cotton, and employ them united in the treatment of Ophthalmia. His method of applying this "quasi specific" is as follows:

"The patient's head having been thrown well back, the surgeon, or an intelligent assistant, opens the eyelids fully, everts them if necessary, and keeps them widely separate. A second person then sets about filling the space of separation with calomel in powder, sprinkling it over the affected part with a brush. The same assistant, or, what is more expeditious, a third person, places immediately over this powder and between the eyelids a roll of cotton, about an inch in length, and two or three lines in diameter. In order to accomplish this last part of the operation conveniently, the two ends of the piece of cotton are laid hold of with the finger and thumb of each hand, and slightly twisted between the fingers. These are then carried to the two angles of the lids; and then both the cotton and the calomel are placed immediately in contact with the membrane of the globe of the eye, and that which lines the eyelids."

The stuffing may be renewed once or twice in the twenty-four hours; and so little pain does it occasion, that even infants submit to the process without repugnance. M. Mayor has seen obstinate ophthalmia yield to this treatment in a day's time.

We find one of the longest memoirs headed "On the rapid cure of Anchylosis:" an attempt not to bolster up merely, but positively to laud the abominable system of treatment advocated by M. Louvrier, which we have already condemned in no measured terms in this Journal. But here we really feel constrained to take leave of our author. It were neither comely nor profitable to take chapter after chapter, and phrase after phrase, for the sole purpose of abuse and condemnation. Our reading of this work was begun with high expectations; and we have sustained proportionate disappointment. We hoped to have given both a full and favorable review of it; but for reasons too obvious and many, we have found ourselves unable to give either. Where we cannot approve, even in some degree, we prefer to be altogether silent.

---

